# The Sanatorium Treatment of Consumption

**Published:** 1904-03-12

**Authors:** T. N. Kelynack

**Affiliations:** Physician to the Mount Vernon Hospital for Consumption and Diseases of the Chest, Hampstead and Northwood, London.


### Hospital Clinics and Medical Progress.

### THE SANATORIUM TREATMENT OF CONSUMPTION.

By T. N. Kelynack, M.D., M.R.C.P., Physician to the Mount Vernon Hospital for Consumption and Diseases of the Chest, Hampstead and Northwood, London.

(*Concluded from page* 405.)

### The After Care of Sanatorium Cases.

It is not my intention to attempt any analysis of the results of sanatorium treatment, although many interesting communications have recently been published on this subject.[23] But I wish, however, to draw attention to the very great loss of time, money, and energy which is often entailed by a neglect to secure proper "after care" when the improved, or it may be recovered patient leaves the sanatorium. In only too many instances a return is at once made to the very conditions which led to the development of the tuberculous process. A wisely directed anticipation might well do something to stop this waste and worse. In a recent visit to the Kelling open-air sanatorium, the admirable institution which Dr. Burton-Fanning of Norwich, with his cousin Mr. W. J. Fanning, as resident medical officer have developed for the consumptive poor of East Anglia, I found serious effort had been made to attack this difficulty. A circular has been prepared which is sent to the friends of the patient, and since it may doubtless prove of interest and value to others engaged in like work, I need make no apology for quoting it in full :—

"It is important to remember that the open-air treatment of the patient should not cease on leaving the sanatorium. Whether the disease is arrested or the condition merely improved it is essential that he should always continue the principles of the treatment. All the living rooms should be abundantly supplied with fresh air, and windows should be kept open day and night. If the patient is to maintain his health he must be well fed and lead a healthy life. This may entail the necessity of changing his former occupation.

"As all this may be difficult to arrange, it is very necessary for the friends and for those interested to begin to consider the question at once and to begin to find suitable employment as soon as possible after the patient's admission.

"The moral effect on the patient of knowing that he has some work to look forward to is very good, and, on the other hand, it is most undesirable that the patient, on leaving the sanatorium, should be met with the anxiety of obtaining work and of providing for himself and his dependents.

"Any employment which does not demand arduous labour or strain and which allows of a good deal of fresh air is suitable ; exposure to bad weather is not necessarily harmful, but work in unventilated rooms or shops is most prejudicial. Light farm work or light gardening work, such clerical work as can be performed in good air, agency work, driving, caretaking—in fact, any light work in good air or out of doors is suitable, but before deciding it will be well to ask advice at the sanatorium, as the exact condition of the patient must be allowed to determine the precise nature of the work for which he is best fitted. This advice will most willingly be given, but the onus of finding suitable employment must rest with the patient's friends."

A little consideration will clearly show that by forethought, co-operation, and what I may call a methodised practical sympathy much more than is usually even attempted might be satisfactorily accomplished.

### Anticipations.

It is difficult to foresee clearly the direction which the development of institutional treatment of consumption is likely to take to this country. Sanatoria of all kinds are rapidly springing into being. Much is being accomplished by private munificence. We need only remember such foundations as our own at Northwood, the King's Sanatoria at Midhurst, Mr. Crossley's Sanatorium in Delemere Forest, and the establishment for the consumptives of Liverpool and district. Much activity is manifest in the development of sanatoria under municipal, county, or other definitely systematised collective action. I have recently visited the very interesting Westmorland Sanatorium, and somewhat similar institutions have been or are being provided for Durham, Norfolk, Nottingham and Worcestershire, Devon and Cornwall, and others are projected for Dorset, Staffordshire and other parts of the country.[24]

Boards of Guardians in many districts are improving their means for providing adequate hygienic treatment of consumptive cases ; but it is most desirable, I venture to think, that sanatoria should be, as far as possible, unconnected with Poor-law administration, at least as now generally conducted. The main difficulty of dealing with the present situation arises when the large number of so-called respectable poor come to be considered. It seems almost impossible for private enterprise to conduct efficiently sanatoria at such prices as shall be within the reach of these needy ones. Attempts have been made and failure has resulted.[25] Further interesting experiments in this direction are now being arranged for. Several private sanatoria are trying to meet the needs of necessitous cases, but the difficulties at present are considerable. I am afraid it must be sorrowfully admitted that present-day philanthropy is altogether inadequate to meet the

[23] See articles by Neissen in *Zeit. fur Tuberk. u. Heilstätt.* Bd. IV., Hf. 2 ; H. Hyslop Thomson in *The Hospital*, October 31st, 1903 ; and F. W. Burton-Fanning and W. J. Fanning in *Lancet*, August 15th, 1903

[24] See *Lancet*, August 15th, 1903, p. 437.

[25] The house established to accommodate 12 men at 17s. a week has had to be closed, *British Medical Journal*, March 7th, 1903.

requirements of the situation. Institutions such as our own which take free cases are overwhelmed with applications. Of the specially selected cases, we have, I am told, about 250 patients waiting for admission.

With Mr. Morton's assistance I have endeavoured to form a rough estimate of the accommodation in sanatoria available for English consumptives.[26] Mr. Morton estimates that there are at least 150,000 consumptive persons in England and Wales, and only about 1,700 beds in sanatoria and consumptive hospitals, including the accommodation in high-class sanatoria.[27] It seems impossible to state definitely the accommodation afforded by sanatoria in this country, because there is no definite standard on which a computation may be based, and, unfortunately, there is, at present, no system of registration and official inspection.

Dr. Bulstrode has recently formulated an interesting calculation. He finds that, excluding Poor-law institutions and including hospitals for consumption and homes for the dying, there would appear to be approximately about 60 institutions in the British Isles devoted mainly, if not entirely, to the care of the consumptive. "The number of beds which are embraced by these institutions is probably under 2,500, and assuming each bed is occupied for three months there may be in England and Wales, Ireland and Scotland, some 10,000 beds available annually. If to this number the accommodation available at workhouses and infirmaries be added the total must be materially augmented."[28]

As regards London consumptives something may be expected from the movement in favour of handing cases over to the care of the Metropolitan Asylums Board. For the wealthy and well-to-do there is no difficulty in finding suitable sanatoria either at home or abroad. In visiting the many establishments in this country I have learnt that in most instances the number of inmates have been much below the desires of the proprietors. The supply of sanatoria for the rich has already considerably outrun the demand. It is clear that many of the so-called sanatoria now in use are far from supplying all the hygienic necessities for effectual treatment. In the conduct of the ordinary routine of these institutions much diversity exists in respect to methods of investigation, manner of treatment, and form of procedure in tabulating results. Without suggesting any desirability for a non progressive uniformity in action, it is much to be desired that some means of scientific co-operation should be arranged for.

In Germany, as is generally known, the State Sick Insurance Fund renders much assistance in the establishment and maintenance of sanatoria for the working class; and efforts have been made to interest the National Friendly Societies in taking similar action in this country.[29] Much activity still continues to be manifest in almost all civilised countries in establishing sanatoria.[30] The interesting movement to establish hygienic homes as a kind of half-way house between the sanatorium and the private dwelling is still only in its beginnings, but wisely directed is capable of much beneficial development.

Much uncertainty still exists as to the exact influence of a marine climate on the tuberculous process. It has been proposed to establish floating sanatoria, and although the disadvantages are evident, some advantages might readily be enumerated[31] In the energy to establish sanatoria for the arrest of pulmonary tuberculosis there has been a tendency to forget the necessity of providing hygienic homes for advanced cases. Pulmonary tuberculosis is no doubt in many cases an eminently curable disease. Statistics are often quoted to prove that it is a rapidly disappearing affection. It has even been suggested that it may become as rare as leprosy within the next few generations. All this is an extravagant and unscientific optimism which is in danger of causing such a reaction as shall give rise to a paralysing pessimism. As Dr. Tatham has well pointed out our statistical returns must be interpreted with much discrimination and circumspection.

Recent pathological researches go far to show that while tuberculosis has a much greater tendency to undergo obsolescence than was formerly believed, it is also much more prevalent than was imagined. Consumption is said to be increasing in Ireland. Certainly such conditions as intemperance, bad feeding, and improper housing are fruitful factors in the causation of consumption, and are widely prevalent. During the past summer I visited all the sanatoria for consumption in Ireland and found that while the available accommodation for well-to-do cases seemed sufficient, the means for adequately dealing with the consumptive poor are miserably meagre.

In attempting any anticipation the outlook must be of the widest. The remarkable awakening in the recent past and the indefatigable industry of many workers in the conduct of the anti-tuberculous campaign in the present, afford the surest security for a continued advance in the near future.

It seems likely, however, that there may be a considerable shifting in opinions and much modification in methods. But be that as it may, there is strong opinion in many quarters entitled to respect, that contagion has been much overrated as a genetic influence, and that lasting progress will be best secured by providing such conditions of life as shall develop the natural resisting powers of the human soil. Comparatively few, I venture to think, share the belief of von Behring that a form of "vaccination" against tuberculosis may ultimately render sanatoria unnecessary. It is well, however, to realise that tuberculosis is not to be exterminated solely by institutions. Sanatoria as remedial establishments have accomplished much, and will continue to bring benefit to many sufferers; as prophylactic institutions they may well be widely extended, and will be of incalculable service; but the chief benefit to the community will prove to be in their educational influence on the thought and life of the people. We do well to strive for cure, arrest, alleviation, but the ultimate goal must be prevention.

[26] *The Hospital*, April 25th, 1903. See also: "Public Health Authorities in relation to the Struggle against Tuberculosis in England." By Arthur Newsholme, M.D., F.R.C.P. *The Journal of Hygiene*, Oct. 1903.

[27] *Medical Press and Circular*, April 29th, 1903, p. 445.

[28] *Lancet*, August 15th, 1903, p. 442.

[29] See *British Medical Journal*, February 14th, 1903; and *Tuberculosis*, London, April, 1903.

[30] See Dr. H. T. Bulstrode's Milroy Lectures "On the Causes, Prevalence, and Control of Pulmonary Tuberculosis," *Lancet*, July 11th, 25th, August 1st, 8th, 15th, 1903.

[31] *British Medical Journal*, January 17th, 1903.

